# Relevance of vitamin D on NAFLD and liver fibrosis detected by vibration controlled transient elastography in US adults: a cross-sectional analysis of NHANES 2017–2018

**DOI:** 10.1080/07853890.2023.2209335

**Published:** 2023-05-08

**Authors:** Yuan Ji, Chang-Bao Wei, Wei Gu, Lin-Lin Hou

**Affiliations:** a Health Management Center, Wuxi People’s Hospital Affiliated to Nanjing Medical University, Wuxi, China; b Department of Joint Surgery and Sports Medicine, Wuxi 9th People’s Hospital Affiliated to Suzhou Medical College of Soochow University, Wuxi, China

**Keywords:** Vitamin D, non-alcoholic fatty liver disease, liver fibrosis, NHANES (National Health and Nutrition Examination Survey)

## Abstract

**Background:**

The connection between vitamin D to non-alcoholic fatty liver disease (NAFLD) is still unclear. Herein, the relationship of vitamin D with NAFLD and liver fibrosis (LF) detected by vibration controlled transient elastography was investigated in US adults.

**Methods:**

The National Health and Nutrition Examination Survey of 2017–2018 was employed for our analysis. Participants were categorized as having either vitamin D deficiency (<50 nmol/L) or vitamin D sufficiency (≥50 nmol/L). A controlled attenuation parameter score of ≥ 263 dB/m was employed to define NAFLD. Significant LF was identified by the liver stiffness measurement value of ≥ 7.9 kPa. Multivariate logistic regression was adopted to explore the relationships.

**Results:**

Among the 3407 participants, the prevalence of NAFLD and LF was 49.63% and 15.93% respectively. Compared to participants without NAFLD, no significant difference in serum vitamin D was observed in NALFD participants (74.26 vs. 72.24 nmol/L; *p* = 0.21). Using multivariate logistic regression analysis, no obvious connection of vitamin D status to NAFLD (sufficiency vs. deficiency, OR 0.89, 95%CI 0.70–1.13) was discovered. However, among NAFLD participants, the sufficiency of vitamin D represents a lower LF risk (OR 0.56, 95%CI 0.38–0.83). When evaluated in quartiles, in comparison to the lowest quartile, high vitamin D represents low LF risk in a dose-dependent manner (Q2 vs. Q1, OR 0.65, 95%CI 0.37–1.14; Q3 vs. Q1, OR 0.64, 95%CI 0.41–1.00; Q4 vs. Q1, OR 0.49, 95%CI 0.30–0.79).

**Conclusions:**

No relationship was found between vitamin D and CAP-defined NAFLD. However, a positive connection of the high serum vitamin D to the reduced LF risk was found among NAFLD subjects.

**Key messages:**
1. Our study found no relationship between vitamin D and CAP-defined NAFLD in US adults.
2. High serum vitamin D was inversely associated with liver fibrosis in a dose-dependent manner among NAFLD participants.

## Introduction

As a most frequently identified liver disorder worldwide, non-alcoholic fatty liver disease (NAFLD) affects about a quarter of the whole population [1]. Considering the epidemic of obesity and diabetes, NAFLD prevalence is projected to continue increasing. NAFLD is considered as a widely types of liver disorder, including simple liver steatosis and non-alcoholic steatohepatitis, which can potentially progressed to liver cirrhosis and fibrosis [2]. NAFLD is characterized as a metabolic disease associated with liver, and frequently accompanied by hypertension, dyslipidaemia, insulin resistance, and obesity. Thus, the NAFLD is renamed as metabolic dysfunction-associated fatty liver disease (MAFLD) [3]. For the quantitation and diagnosis of fatty liver, biopsy is the gold standard. However, because of the possible sampling error, elevated costs, and invasive nature, it is unsuitable to use it in a broad population. Thus, in population studies, the serum biomarkers and ultrasonography are frequently employed. However, ultrasonography has limited sensitivity and does not reliably detect steatosis when the degree of steatosis <20% [4]. As a non-invasive and accurate method, the vibration controlled transient elastography (VCTE) is frequently employed to examine liver steatosis and fibrosis severity of NAFLD patients, and simultaneously record the values of controlled attenuation parameter (CAP) and liver stiffness measurement (LSM) [5]. Meanwhile, it is also commonly used for the evaluation of hepatic fibrosis and steatosis in general population [6].

Vitamin D is a sterol hormone synthesized in the skin through a chemical reaction dependent on ultraviolet radiation and is later activated by hydroxylation in liver and kidneys. Except its classical functions in regulating calcium homeostasis and the bone metabolism [7], vitamin D has extensive extra-skeletal effects such as anti-inflammatory [8], anti-fibrotic [9], and immunomodulatory [10] properties. Vitamin D deficiency, characterized by low 25-hydroxy vitamin D (25(OH)D) in serum, has been correlated with increasing risks of cardiovascular diseases [11], metabolic syndrome [12], and type 2 diabetes [13]. The contradictory results about the association between NAFLD and the deficiency of vitamin D were obtained previously [14–16]. In a health check-up Korean population, connection of the high serum vitamin D to the reduced NAFLD risk was observed by Heo et al. [14]. However, another study in South Korea found that serum vitamin D concentration is not connected to MAFLD [15]. A recent bi-directional mendelian randomization study also supported no association between low vitamin D and ultrasound-diagnosed NAFLD [16].

Therefore, according to the 2017–2018 National Health and Nutrition Examination Survey (NHANES), we conducted this cross-sectional study to examine the connection of serum vitamin D to NAFLD and liver fibrosis (LF) detected by VCTE, providing novel ideal for liver steatosis and fibrosis diagnosis at an early stage.

## Methods

### Study population

As a survey program based on national population, NHANES is performed every 2 years and used to evaluate the nutritional and health status of the non-institutionalized US civilian general population. Herein, we employed the NHANES of the 2017–2018 cycle for our analysis because of the data of VCTE was specifically included.

**Figure 1.**
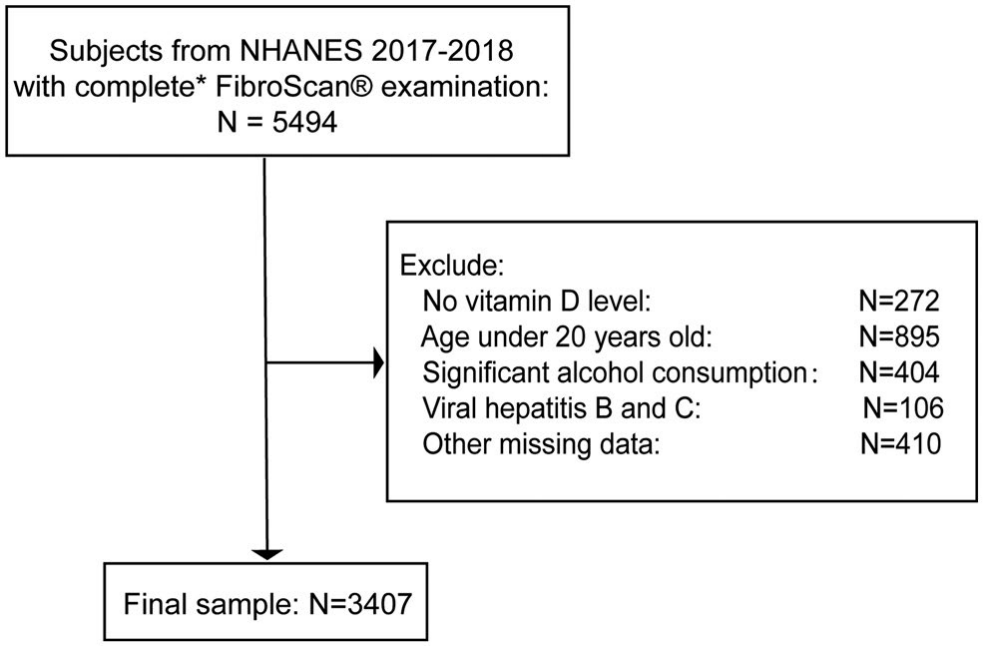
Flow chart for study population selection. *Complete transient elastography (FibroScan®) examination was defined as a fasting time of ≥3 h, ≥10 complete liver stiffness measures, and a liver stiffness interquartile range/median <30%.

A total of 9254 participants were enrolled in this cycle. Among them, 5494 participants had elastography exam with complete status. We excluded 272 participants with missing vitamin D data and 895 participants aged below 20 years. For NAFLD, 404 participants with significant alcohol intake (>20 g/day for females and >30 g/day for males) were excluded [17]. We also excluded 106 hepatitis B or C-infected participants (characterized as the presence of hepatitis C antibody or the hepatitis B surface antigen) and 410 participants with other missing data. Finally, we enrolled 3407 participants in our present study (Figure 1). According to the hepatic steatosis determined by CAP and any of the three metabolic conditions, such as diabetes mellitus, overweight/obesity, or metabolic dysregulation, MAFLD was identified [3].

The original NHANES study protocol was approved by the Ethics Review Board of the National Center for Health Statistics Research. The signed written informed consents were provided by all the enrolled individuals.

### Measurement of serum vitamin D

In our present study, the total 25(OH)D in serum was set as the concentration of 25(OH)D2 plus 25(OH)D3. For measuring the 25(OH)D concentrations, the high-performance liquid chromatography-tandem mass spectrometry (HPLC-MS/MS) was conducted by the National Center for Environmental Health. According to the 25(OH)D concentrations in serum, we separated the participants to two groups: the vitamin D deficiency group, whose participants had less than 50 nmol/L vitamin D in serum and the vitamin D sufficiency group, whose participants had no less than 50 nmol/L vitamin D in serum [14,18]. In addition, we also defined the quartile 1, 2, 3, and 4 of vitamin D in serum as 9.96–47.05 nmol/L, 47.05–65.00 nmol/L, 65.00–85.65 nmol/L, and 85.65–372.00 nmol/L, respectively. The vitamin D level quartile 1 was employed as the reference group.

### Vibration controlled transient elastography (VCTE)

The examination of transient elastography was carried out to record the data and diagnose NAFLD and LF. A XL or M probe equipped FibroScan® model 502 V2 Touch (Echosens, Paris, France) was employed to conduct the elastography exam in the NHANES Mobile Examination Center. For each participant, no less than 10 measurements were obtained, and the values of median CAP and LSM were calculated by the device along with the interquartile range. Exams were considered complete if participants fasted at least 3 h before the exam, there were 10 or more complete liver stiffness measures, and the interquartile range/median of the LSM was < 30%. In this study, CAP value of 263 dB/m or higher was used to define NAFLD [19,20] and we also set the LSM ≥ 7.9 kPa as significant LF [21].

### Covariates

Based on the previous report [22], the NAFLD-associated covariates, such as total cholesterol (TC), high-density lipoprotein cholesterol (HDL-C), estimated glomerular filtration rate (eGFR), diabetes, hypertension, body mass index (obesity: ≥30 kg/m^2^, overweight: 25–30 kg/m^2^, and under/normal weight: < 25 kg/m^2^), smoking status (never smoker, former smoker, or current smoker), education level (> high school or ≤ high school), race/ethnicity (Asian, Mexican-American, Non-Hispanic Black, Non-Hispanic White, and Other Races), sex, and age, were included. Dietary vitamin D intake and supplemental vitamin D intake were also adjusted to account for the potential impact of dietary factors. We computed eGFR using the Chronic Kidney Disease Epidemiology Collaborative equation [23]. Diabetes was defined by the following criteria: no less than 6.5% haemoglobin A1c level, self-reported diagnosis of diabetes, or taking diabetic medications. The participants with more than 80 mmHg diastolic blood pressure, or more than 130 mmHg systolic blood pressure, or taking hypertension medications, or high blood pressure told by a doctor, were defined as hypertension [24].

### Statistical analysis

Continuous variables were expressed as mean ± SE and categorical variables were expressed as number and percentage. The two sample *t*-test and chi-square test were carried out for comparing the continuous and categorical variables between groups, respectively. For exploring the connections of vitamin D to NAFLD and LF, we conducted the multivariate logistic regression analyses. Three different logistic models, such as non-adjusted model 1, ethnicity-, sex-, and age-adjusted model 2, and TC-, HLD-C-, eGFR-, diabetes-, hypertension-, BMI group-, dietary vitamin D intake-, supplemental vitamin D intake-, smoking status-, education level-, and model 2 covariates-adjusted model 3, were employed in our present study. As suggested by NHANES, sample weights used for accounting for unequal selection probabilities and nonresponse were utilized in all analyses. Statistical analysis was conducted with R 4.2.0 software and the value of *p* less than 0.05 was considered statistically significant.

## Results

### Characteristics of study population

According to the exclusion criteria, we enrolled 3407 participants in this analysis. The mean age of our study population was 48.54 ± 0.79 years, and 47.78% of the participants were male. The weighted prevalence was 49.63% for NAFLD, and the weighted prevalence of significant fibrosis among NAFLD participants was 15.93%. Mean serum vitamin D level was 73.25 ± 1.66 nmol/L, with 21.25% of participants below 50 nmol/L. Mean dietary vitamin D intake and supplemental vitamin D intake were 4.24 ± 0.12 mcg/day and 21.82 ± 2.81 mcg/day, respectively. Based on the status of vitamin D, Table 1 summarizes the clinical characteristics of participants. The vitamin D deficient participants were younger and had lower education level. They showed higher BMI and lower HDL-C levels. They also had less dietary and supplemental intakes of vitamin D. Between the participants with deficient and sufficient vitamin D, no significant difference in the prevalence of hypertension, diabetes, and NAFLD defined by CAP ≥ 263 dB/m were discovered. In comparison to vitamin D sufficient participants, high percentage of participants with LSM ≥ 7.9 kPa was observed in the vitamin D deficiency group.

**Table 1.** Characteristics of the study population according to vitamin D status.

Characteristics	Vitamin D Deficiency (<50 nmol/L) (*n* = 966)	Vitamin D Sufficiency (≥50 nmol/L) (*n* = 2441)	*p* ^a^
Age (years)	41.98 ± 0.65	50.31 ± 0.79	<0.0001
Male, n (%)	481 (52.29)	1147 (47.12)	0.06
Race/Ethnicity, n (%)			<0.0001
Non-hispanic white	146 (34.69)	1045 (70.75)	
Non-hispanic black	389 (27.27)	378 (6.35)	
Mexican American	167 (16.84)	313 (6.91)	
Asian	132 (7.89)	334 (4.84)	
Other race	132 (13.30)	371 (11.16)	
Education level, n (%)			0.002
≤High school	439 (45.61)	1014 (36.17)	
>High school	527 (54.39)	1427 (63.83)	
Smoking status, n (%)			0.02
Never smoker	603 (62.85)	1426 (59.08)	
Former smoker	173 (19.15)	656 (26.69)	
Current smoker	190 (18.00)	359 (14.24)	
BMI (kg/m^2^)	31.15 ± 0.46	29.50 ± 0.31	<0.001
BMI group, n (%)			<0.001
Underweight/normal (<25)	219 (22.33)	642 (26.58)	
Overweight [25–30]	262 (26.43)	837 (31.50)	
Obesity (≥30)	485 (51.24)	962 (41.92)	
Vitamin D (nmol/L)	37.13 ± 0.41	83.00 ± 1.16	<0.0001
ALT (U/L)	24.10 ± 1.02	22.10 ± 0.42	0.12
AST (U/L)	22.17 ± 0.73	21.18 ± 0.29	0.27
HDL-C (mg/dL)	49.29 ± 0.60	53.60 ± 0.61	<0.0001
TC (mg/dL)	184.97 ± 2.73	190.67 ± 2.05	0.04
eGFR (mL/min/1.73 m^2^)	104.20 ± 0.99	90.76 ± 0.96	<0.0001
Hypertension	535 (49.47)	1470 (53.01)	0.18
Diabetes	185 (15.43)	525 (15.25)	0.92
Dietary vitamin D intake (mcg/day)	3.45 ± 0.14	4.46 ± 0.15	<0.001
Supplemental vitamin D intake (mcg/day)	3.29 ± 1.07	26.82 ± 3.53	<0.0001
CAP (dB/m)	270.42 ± 3.78	262.94 ± 2.24	0.11
CAP ≥ 263, n (%)	509 (53.61)	1257 (48.56)	0.08
LSM (kPa)	6.15 ± 0.28	5.60 ± 0.09	0.06
LSM ≥ 7.9, n (%)	119 (13.69)	246 (8.51)	0.001

Data are presented as mean ± SE for continuous variables.

^a^Two sample t-test was performed for continuous variables, and chi-square test was performed for categorical variables.

BMI, body mass index; ALT, alanine aminotransferase; AST, aspartate aminotransferase; HDL-C, high-density lipoprotein cholesterol; TC, total cholesterol; eGFR, estimated glomerular filtration rate; CAP, controlled attenuation parameter; LSM, liver stiffness measurement.

### Association of vitamin D level with NAFLD/MAFLD

In comparison to individuals without NAFLD, no significant difference in serum vitamin D was observed in NALFD participants (74.26 ± 1.30 vs. 72.24 ± 2.25 nmol/L; *p* = 0.21). After adjusting for TC, HDL-C, eGFR, diabetes, hypertension, BMI group, dietary vitamin D intake, supplemental vitamin D intake, smoking status, education level, race/ethnicity, sex, and age, no significant associations of vitamin D status or quartiles with NAFLD were observed in the multivariate logistic regression analysis. Among the enrolled 3407 subjects, 48.42% of the participants were MAFLD. Additionally, the correlation of vitamin D with MAFLD were similar to that of vitamin D with NAFLD (Table 2).

**Table 2.** Multivariate analysis for the association between vitamin D and NAFLD/MAFLD.

Vitamin D	NAFLD		MAFLD	
	OR (95% CI)	*p*	OR (95% CI)	*p*
Sufficiency vs. Deficiency	0.89 (0.70, 1.13)	0.30	0.84 (0.64, 1.10)	0.18
Quartile				
Q1 (9.96, 47.05)	1 (reference)		1 (reference)	
Q2 (47.05, 65.00)	0.91 (0.70, 1.19)	0.49	0.85 (0.65, 1.12)	0.23
Q3 (65.00, 85.65)	0.76 (0.54, 1.06)	0.10	0.75 (0.53, 1.07)	0.10
Q4 (85.65, 372.00)	0.79 (0.49, 1.27)	0.31	0.74 (0.41, 1.31)	0.28

OR (95% CI), odds ratio (95% confidence interval); NAFLD, non-alcoholic fatty liver disease; MAFLD, metabolic dysfunction-associated fatty liver disease.

Values are adjusted for age, sex, race/ethnicity, education level, smoking status, body mass index group, hypertension, diabetes, high-density lipoprotein cholesterol, total cholesterol, estimated glomerular filtration rate, dietary intake of vitamin D, and supplement intake of vitamin D.

### Association of vitamin D level with LF in NAFLD participants

Among the subset of people with NAFLD, significantly fibrotic participants showed low vitamin D concentrations in the serum (67.40 ± 2.97 vs. 73.15 ± 2.20, *p* = 0.01). For evaluating the adjusted connection of vitamin D level to LF, the multivariate logistic regression analysis was conducted. In the unadjusted model, vitamin D status (sufficiency vs. deficiency) was inversely correlated to LF (OR 0.56, 95%CI 0.41–0.78). And this inverse association was also present in adjusted model 2 (OR 0.49, 95%CI 0.33–0.72) and 3 (OR 0.56, 95%CI 0.38–0.83). When we evaluated vitamin D levels in quartiles, a dose-dependent relationship between LF and higher vitamin D levels was observed in comparison to those with lowest quartile after adjusting multiple confounding factors (Q2 vs. Q1, OR 0.65, 95%CI 0.37–1.14; Q3 vs. Q1, OR 0.64, 95%CI 0.41–1.00; Q4 vs. Q1, OR 0.49, 95%CI 0.30–0.79) (Table 3).

**Table 3.** Multivariate analysis for the association between vitamin D and liver fibrosis.

Vitamin D	Model 1		Model 2		Model 3	
	OR (95% CI)	*p*	OR (95% CI)	*p*	OR (95% CI)	*p*
Sufficiency vs. Deficiency	0.56 (0.41, 0.78)	0.002	0.49 (0.33, 0.72)	0.003	0.56 (0.38, 0.83)	0.01
Quartile						
Q1 (9.96, 47.05)	1 (reference)	0.002*	1 (reference)	0.004*	1 (reference)	0.01*
Q2 (47.05, 65.00)	0.68 (0.40, 1.14)	0.13	0.63 (0.34, 1.15)	0.11	0.65 (0.37, 1.14)	0.12
Q3 (65.00, 85.65)	0.66 (0.41, 1.06)	0.08	0.57 (0.32, 1.01)	0.05	0.64 (0.41, 1.00)	0.05
Q4 (85.65, 372.00)	0.47 (0.31, 0.71)	0.002	0.37 (0.21, 0.64)	0.005	0.49 (0.30, 0.79)	0.01

OR (95% CI), odds ratio (95% confidence interval);.

Model 1: no covariates were adjusted.

Model 2: age, sex, and race/ethnicity were adjusted.

Model 3: age, sex, race/ethnicity, education level, smoking status, body mass index group, hypertension, diabetes, high-density lipoprotein cholesterol, total cholesterol, estimated glomerular filtration rate, dietary intake of vitamin D, and supplement intake of vitamin D, were adjusted.

* *p* for trends.

## Discussion

Using a large general US population, our study found that serum vitamin D is not connected to the CAP-defined NAFLD, however, among NAFLD participants, low vitamin D represents high risk of developing significant LF.

The contradictory conclusions of the correlation between NAFLD and vitamin D have been obtained from several studies [16,25–29]. According to the data from NHANES III, previous studies concluded that relatively low vitamin D in serum is significantly connected to elevated NAFLD [25] or MAFLD risk [26]. In their studies, hepatic steatosis was confirmed by ultrasonography, and their mean vitamin D levels were lower than the levels of our study. Similar to our result, using the data from the Korea NHANES, researchers also observed similar vitamin D concentrations in serum from 409 NAFLD subjects and 1403 control subjects without NAFLD [27]. Similarly, no significant association of the reduced vitamin D with the hepatic steatosis was found in general Portuguese population [28]. Apart from conventional case-control and cross-sectional studies, other researchers conducted bidirectional Mendelian randomization study, which used genetic variants in non-experimental data to make causal inferences and could avoid problems in conventional epidemiological studies such as reverse causation and residual confounding. Using three European descent populations, the researchers observed an association of genetically predicted higher vitamin D levels with the reduced NAFLD risk [29]. However, another Mendelian randomization study using 4568 healthy controls and 4614 NAFLD subjects from east Chinese population found that the four genetic variants-instrumented vitamin D is not associated with the NAFLD risk [16]. In an animal study, deficient vitamin D could even cause alleviation of hepatic lipid accumulation induced by high-fat diet through inhibiting hepatic *PPARγ* and up-regulating carnitine palmitoyltrans 2, the major enzyme for fatty acid *β*-oxidation [30].

As a hallmark characteristic of liver injury, the advanced LF can comprise severe risks for development of hepatocellular carcinoma and hepatic failure. In consistent with our current conclusion, the negative correlation of vitamin D with LF determined by NAFLD fibrosis score was observed by other researchers [18,31]. In the biopsy-proven NAFLD patients, Arai et al. also demonstrated an inversely association of advanced LF with vitamin D [32]. However, a study based on NHANES III revealed that advanced fibrosis identified by non-invasive scores are not connected to the low 25(OH)D in serum [26]. The inconsistent conclusions may result from the different measurements of LF and various vitamin D concentrations.

Currently, why vitamin D could effectively suppress the LF in NAFLD patients remains unclear. During the development of LF, hepatic stellate cell activation plays important effect. Activated hepatic stellate cells can effectively induce cell growth and transformation from a quiescent vitamin A-storing cell into an activated myofibroblast-like cell, increasing the production of extracellular matrix and tissue inhibitors of metalloproteinases [33]. Through inhibiting the activation of TGF-β/Smad signalling pathway in hepatic stellate cells, vitamin D can effectively impair the fibrosis of liver [34]. A recent study further indicated that vitamin D reduced hepatic stellate cells and TGF-β/Smad signalling activation through negative regulation of histidine-rich calcium binding protein [35].

No effective treatment strategies for hepatic fibrosis are available so far. Based on previous research, we believe that the supplementation of vitamin D may be used as new widely available and cost-effective treatment for LF prevention and therapy. A randomized double-blind placebo-controlled trial, including 311 NAFLD patients, found that 12-month usage of vitamin D (1000 IU/day) can significantly attenuate the transient elastography indices of liver steatosis and fibrosis [36]. Another trial failed to show an effect of vitamin D on the hepatic fat fraction measured by magnetic resonance and on biochemical markers of fibrosis after 6 months treatment of type 2 diabetes patients with NAFLD [37]. Nevertheless, for complete revealing the regulation of vitamin D in the fibrogenesis of liver and assessing the efficiency and safety of vitamin D for LF treatment in NAFLD patients, further trials with longer term are warranted.

The strength of this study is that a nationally representative data is included. Using this dataset, we could analyse the data from healthy individuals and are not restricted to certain kinds of patients such as diabetes. However, there are also some limitations present in our study. First, due to the cross-sectional design, the verification of causality was limited, and the impact of vitamin D over time was not evaluated. Second, there are no consensual cut-offs for CAP and LSM. Third, the vitamin D seasonal variations, as well as the usage of supplement containing calcium were not investigated in this study.

## Conclusions

In summary, based on a large general US population, this study demonstrated that the CAP-defined NAFLD is not associated with the deficiency of vitamin D. On the other hand, low serum vitamin D was closely connected to increased LF risk among NAFLD participants. Thus, the role of vitamin D in prevention and treatment of NAFLD warrants further research.

## Data Availability

The survey data are publicly available on the internet for data users and researchers throughout the world (https://www.cdc.gov/nchs/nhanes/).
